# Predicting *Culex pipiens/restuans* population dynamics by interval lagged weather data

**DOI:** 10.1186/1756-3305-6-129

**Published:** 2013-05-02

**Authors:** Karin Lebl, Katharina Brugger, Franz Rubel

**Affiliations:** 1 Institute for Veterinary Public Health, University of Veterinary Medicine Vienna, Veterinärplatz 1, Vienna, A-1210, Austria

**Keywords:** *Culex pipiens*, *Culex restuans*, Cross-correlation map, Mosquito vector, Population dynamics, Predictive model, Seasonal cycle

## Abstract

**Background:**

*Culex pipiens/restuans* mosquitoes are important vectors for a variety of arthropod borne viral infections. In this study, the associations between 20 years of mosquito capture data and the time lagged environmental quantities daytime length, temperature, precipitation, relative humidity and wind speed were used to generate a predictive model for the population dynamics of this vector species.

**Methods:**

Mosquito population in the study area was represented by averaged time series of mosquitos counts captured at 6 sites in Cook County (Illinois, USA). Cross-correlation maps (CCMs) were compiled to investigate the association between mosquito abundances and environmental quantities. The results obtained from the CCMs were incorporated into a Poisson regression to generate a predictive model. To optimize the predictive model the time lags obtained from the CCMs were adjusted using a genetic algorithm.

**Results:**

CCMs for weekly data showed a highly positive correlation of mosquito abundances with daytime length 4 to 5 weeks prior to capture (quantified by a Spearman rank order correlation of *r*_*S*_ = 0*.*898) and with temperature during 2 weeks prior to capture (*r*_*S*_ = 0*.*870). Maximal negative correlations were found for wind speed averaged over 3 week prior to capture (*r*_*S*_ = *−*0*.*621). *Cx. pipiens/restuans* population dynamics was predicted by integrating the CCM results in Poisson regression models. They were used to simulate the average seasonal cycle of the mosquito abundance. Verification with observations resulted in a correlation of *r*_*S*_ = 0*.*899 for daily and *r*_*S*_ = 0*.*917 for weekly data. Applying the optimized models to the entire 20-years time series also resulted in a suitable fit with *r*_*S*_ = 0*.*876 for daily and *r*_*S*_ = 0*.*899 for weekly data.

**Conclusions:**

The study demonstrates the application of interval lagged weather data to predict mosquito abundances with a feasible accuracy, especially when related to weekly *Cx. pipiens/restuans* populations.

## Background

*Culex pipiens* (Diptera: Culicidae), the northern house mosquito, is widely distributed all over the world except Australia and Antarctica [1]. *Cx. restuans* is rather similar in morphological characteristics and breeding habitat preferences but exclusively distributed in North America [2]. Both are important vectors for arthropod borne viral infections affecting the health of humans, domestic and wild animals. They transmit diseases like West Nile fever, St. Louis encephalitis, Japanese encephalitis, Western equine encephalitis, and Rift Valley fever [1,3,4]. As mosquitoes are poikilothermic animals investigating the *Cx. pipiens/restuans* population dynamics in relation to factors like ambient temperature and rainfall could help to predict the population dynamics of this species, which is an essential requirement for an efficient vector control.

Previous studies have already confirmed that population densities vary strongly with temperature [5,6]. This is due to the temperature dependence of the development rates of eggs, larvae and pupae, survival rates of immatures as well as imagos. Temperature also influences the length of the gonotrophic cycle [7-10]. Furthermore, during winter *Cx. pipiens/restuans* undergo reproductive diapause controlled not only by temperature, but also by daytime length [11,12].

Also precipitation has already been identified as another important factor influencing *Cx. pipiens/restuans* population dynamics, as high rainfall occurring several weeks before a capture event was positively correlated with the mosquito abundance [5,6]. The accurate mechanisms behind this relationship however are less well understood. It has been suggested that rainfall increases the water surface area and therefore possible oviposition sites [6,13]. Precipitation may also negatively affect mosquito capture, as the immature stages might get washed away by heavy rainfall. Geery [14] recorded a partial flushing of larvae (22–34% reduction) from catch basins in Cook County by rain events *<* 25 mm and an up to 91% reduction for strong rainfall of *>* 100 mm. Furthermore, precipitation may also decrease adult activity levels [15,16].

One of the classical approaches to investigate the relation between mosquito population dynamics and weather data is to correlate their time series [13,17]. In 2005 Curriero et al. [18] introduced cross-correlation maps (CCMs) as a tool to study the influence of environmental conditions during a time lagged interval (instead of using point lags, i.e., the conditions at a certain time point prior to the capture event) on the abundance of *Ochlerotatus sollicitans*, another Culicidae species. They demonstrated that lagged time intervals are better adapted to describe the mosquito abundance than point lags. Since then CCMs have been used to illustrate the correlations between various environmental factors and population size of *Aedes sollicitans*, *Ae. vexans*, *Cx. pipiens*, *Cx. restuans* and *Cx. salinarius*[5,19,20].

The aim of this study is to use interval lagged correlations between environmental factors and observed *Cx. pipiens/restuans* abundance, gained from CCMs, to develop a predictive model of mosquito population dynamics. This mosquito model should be able to simulate the seasonal cycle of *Cx. pipiens/restuans* populations and help uncover the temporal scale influences on model performance by comparing daily versus weekly predictions. Such a model would be immensely useful for risk assessment studies i.e. of such arboviral diseases as mentioned above. An analogous study using CCMs and simulated population dynamics of midges was recently presented by the authors for Bluetongue disease in Austria [21]. In this study a mosquito model developed for a spatial scale representative for a major part of Cook County, and not for specific localized capture sites, is introduced. Such models encompassing larger areas are important tools for investigating mosquito born disease outbreaks and their intervention strategies.

## Methods

### ***Cx. pipiens/restuans ***capture data

**Figure 1:**
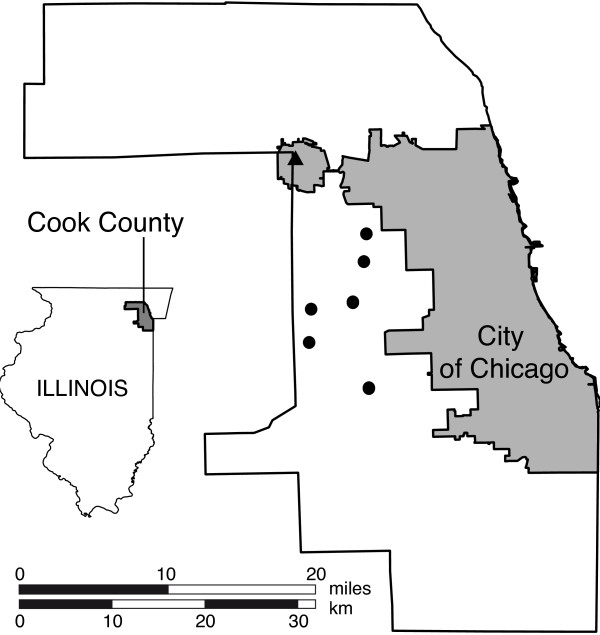
**Location of the 6 trap sites in Cook County, Illinois.** Location of the traps marked with dots, the triangle represents the location of the weather station at the Chicago O’Hare International Airport (WMO No. 72530).

Mosquito count data for Cook County (Illinois, U.S.A.) were obtained from the Desplaines Valley Mosquito Abatement District (P. Geery, 2011, pers. comm.). This agency provides long term data of daily separated capture rates since 1980. Mosquitoes have been captured using 25 New Jersey light traps (NJLT) located in the townships of Lyons, Oak Park, Proviso, Riverside, and River Forest. All traps were located within the suburbs of Chicago, in”medium intensity developed” areas (National Land Cover Database [22]). However, not all trap sites were operated continuously during the entire time span of the study. Trap operation time per site varied from 3 to 31 years. For this study we aimed to generate an average time series of *Cx. pipiens/restuans* catches. To avoid bias due to gaps in the single time series, only continuous series of a minimum length of 20 years were selected. Furthermore, we excluded one site showing inhomogeneity in its time series. The number of female mosquitoes were averaged over the remaining 6 capture sites (Figure 1). Of the 83507 *Culex* females caught by these trap sites between 1991 and 2010, 97% were *Cx. pipiens/restuans*, the rest were *Cx. territans*, *Cx. tarsalis*, *Cx. erraticus* and *Cx. salinarius*. The catches of *Cx. pipiens* and *Cx. restuans* were combined by the mosquito control agency, as their differentiation based on morphological characteristics is rather imprecise [2]. The 6 single trap sites provided 23722, 7945, 6141, 19543, 9468 and 14203 individuals to the total count of 81022 captured *Cx. pipiens/restuans* females.

To create a predictive model valid for the whole year, it was necessary to generate a continuous time series of the capture data. As *Culex* mosquitoes are not active at low temperatures, the mosquito control agency did not operate their traps during winter time. Mosquito counts for winter time, i.e. when no traps were set up, were assumed to be zero (21. Oct. - 30. April). Remaining missing values (6.9%) were replaced by corresponding values of the averaged annual course of the *Cx. pipiens/restuans* catches (averaged over all years and locations).

### Environmental data

The meteorological quantities used in this study were taken from the weather station of the Chicago O’Hare International Airport (WMO No. 72530 [23]) located at geographical longitude *λ* = 87*.*900° W, latitude  = 41*.*983° N and altitude *h* = 205 m. Air temperature *T* in *°*C, precipitation *P* in mm/day, relative humidity *H* in % (calculated from air and dew point temperature) and wind speed *W* in m/s were selected for this study as quantities potentially influencing mosquito abundance. Another quantity, influencing mosquito diapause, is the daytime length *D* in hours. It is a function of the sun’s declination *ϵ* and the geographic latitude and was calculated using the CBM model described by Forsythe [24]:

(1) $$D=24-\frac{24}{\pi }\;\mathrm{acos}\left(\frac{\sin\left(0.8333\right)+\sin\left(\varphi \right)\sin\left(\epsilon \right)}{\cos\left(\varphi \right)\cos\left(\epsilon \right)}\right)$$

with the declination of the sun *ϵ* calculated as a function of the calendar day *d*:

(2) $$\begin{array}{l} \epsilon =\mathrm{asin}(0.39795\;\cos(0.2163108\\ \;+2\;\mathrm{atan}\left(0.9671396\;\tan\left(0.00860\;\left(d-186\right)\right)\right))) \end{array}$$

**Figure 2:**
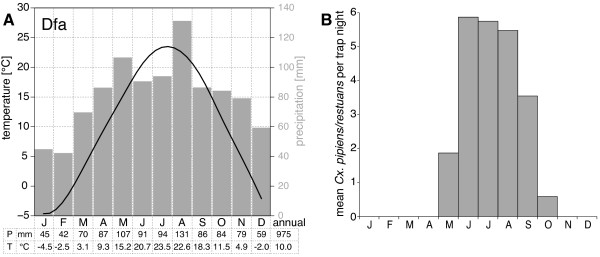
**Seasonal cycle.** Annual mean climatic conditions and mosquito abundances for Cook County (Illinois). **A:** Climate diagram compiled from temperature (line) and precipitation (bars) measurements used in this study. Location Chicago O’Hare international airport, period 1991–2010, Köppen-Geiger climate classification Dfa. **B:** Mean number of female *Cx. pipiens/restuans* per trap night, averaged over the study period of 20 years and 6 trapping sites.

Figure 2 depicts the climate diagram calculated from temperature and precipitation data used in this study. Following the well known Köppen-Geiger climate classification [25], the climate conditions in Cook County were characterized by Dfa climate (D = continental or snow climate, f = fully humid, a = hot summers).

### Statistical methods

Two different time scales were used in the analysis: daily and weekly data, wherefore we calculated the weekly means from the daily data. In a first step cross-correlation maps (CCMs) were applied to determine the maximal correlations between mosquito abundance and environmental quantities. CCMs illustrate the correlation coefficients between *N*_*i*_, the number of captured mosquitoes at time *i* and an environmental quantity *X*, averaged over a time period starting at time *i − j* (time lag 1) and ending at *i − k* (time lag 2), with *j ≥ k*. Thus, a CCM at the coordinates *j* and *k* illustrates

(3) $$\mathrm{CC}M_{j,k}=\mathrm{cor}\left(N_{i},{\bar{X}}_{i-j,i-k}\right)$$

**Figure 3:**
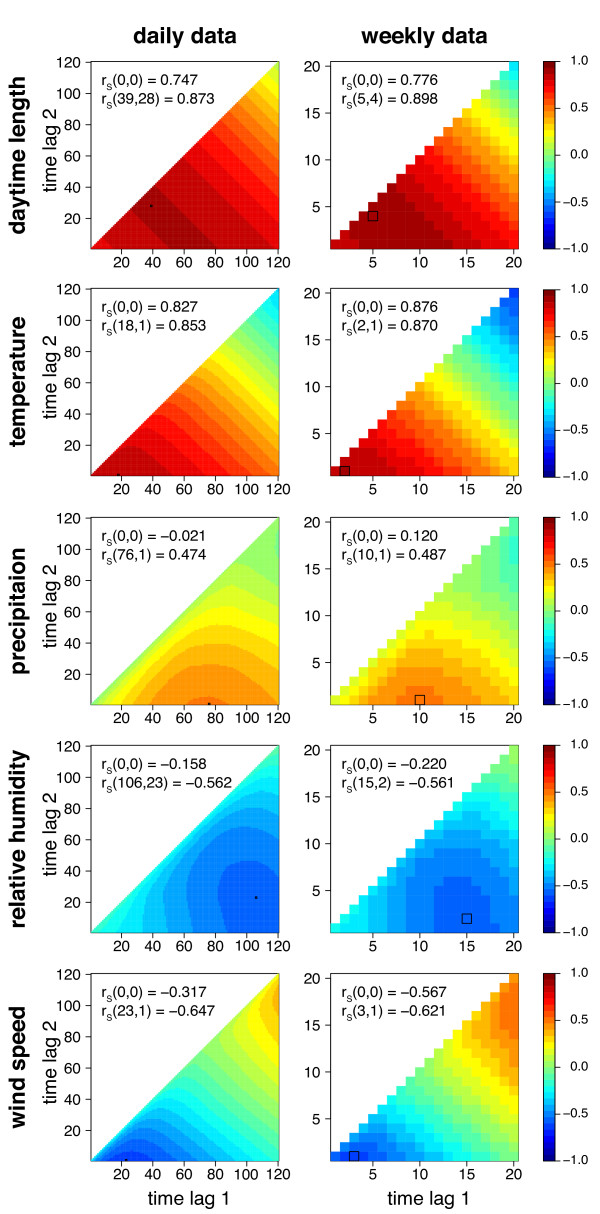
**Cross correlation maps.** CCMs for daily and weekly data. CCMs depict the correlations (Spearman rank order correlation coeffcients) between *Cx. pipiens/restuans* mosquito capture data and daytime length [h], temperature [°C], precipitation [mm], relative humidity [%] and wind speed [m/s]. The correlation coefficient for the day or week of the capture *r*_*S*_ (0*,* 0) as well as the maximum of the lagged correlation coefficient *r*_*S*_ (*lag*1*, lag*2) (black frame) are given.

In the case of j = k (plotted in the diagonal), the correlation coefficients are equal to those of a cross-correlogram. CCMs for the selected environmental quantities daytime length, temperature, precipitation, relative humidity and wind speed are depicted in Figure 3. Spearman’s rank order correlation was applied because mosquito capture rates as well as some environmental quantities, especially daytime length and precipitation, are non-Gaussian distributed. The maximum time lag was set to 120 days and 20 weeks, respectively, to make sure that the maximum correlations were found. The correlation coefficient gained by correlating the environmental quantities at the capture event (*r*_*S*_ (0*,* 0); i.e., no lags were applied) with the mosquito time series (using Spearman’s rank correlation) is a result not only by the effect of those quantities on mosquito abundance, but also from the effects on their trapping probability. Thus, as the CCMs were used to describe the relation between the environmental quantities and the abundance of *Cx. pipiens/restuans*, the environmental quantities at the time of the capture were excluded. However, for the predictive model (see below) the influence of the environmental quantities on the trapping probability is likely to be important to replicate the capture rates.

For the generation of the predictive model the data set was divided into training and test data. To archive a uniform distribution of the test data over the 20 years the data were split in even and odd years. The odd years were selected by a random process to be the test data set.

The interval lagged environmental quantities daytime length (D), temperature (T), precipitation (P), humidity (H) and wind speed (W) were integrated in a Poisson regression model to predict *Cx.pipiens/restuans* abundance:

(4) $$\begin{array}{l} \mathrm{lo}g_{e}\left(N_{i}\right)=\beta_{0}+\beta_{1}D_{i}+\beta_{2}{\bar{D}}_{i-j,i-k}+\beta_{3}T_{i}+\beta_{4}{\bar{T}}_{\;i-j,i-k}\\ \;+\beta_{5}P_{i}+\beta_{6}{\bar{P}}_{\;i-j,i-k}+\beta_{7}\;H_{i}+\beta_{8}{\bar{H}}_{\;i-j,i-k}+\beta_{9}W_{i}+\beta_{10}{\bar{W}}_{\;i-j,i-k} \end{array}$$

In this model the environmental conditions at the capture event (*X*_*i*_) were also included, as they could affect mosquito activity and thus the capture rate. Please note that *j* and *k* for the lags of the five parameters do not necessarily have the same values. Non-relevant terms from this full model were removed in a stepwise procedure to identify the model with the lowest AIC (Akaike’s Information Criterion [26]). The resulting model (*CCM*) was used to predict the mosquito abundance.

However, as the effect of the environmental conditions possibly interact, inclusion of the lags obtained from the CCMs might not result in the best optimal predictive model. Therefore, the next step was to generate a predictive model with a higher fit by identifying the best combinations of lags for the environmental quantities. Again, parameter combinations with less parameters than the full model were considered as well, and model fit was used to evaluate by the AIC. As it was not possible to test all different lag combinations, a genetic algorithm was used to identify the model with the highest fit. Genetic algorithms are heuristic techniques to solve optimization problems by emulating the processes of natural selection [27]. Here, the beginning and end of each time lag from the environmental quantities was subject to this process as well as the decision to include a certain parameter. The generated combination of time lags was implemented in the regression model. The AIC was chosen as the optimization criterium. For the daily data 5000 iterations were conducted, while 2000 were used for the weekly data. For both data sets the population size was set to 200 and the genetic algorithm was run 10 times, showing that all of those runs converged to the same results. The final model from the genetic algorithm with the optimal combinations of lags is called *OPT* (Table 1).

**Table 1:** Parameters of the regression models

**parameter**	**daily**		**weekly**	
	**CCM**	**OPT**	**CCM**	**OPT**
*D*_0*,*0_	+	+	—	+
*D*_*lag*1*,lag*2_	39, 28	85, 85	5, 4	13, 13
*T*_0*,*0_	+	+	+	+
*T*_*lag*1*,lag*2_	18, 1	21, 1	2, 1	1, 1
*P*_0*,*0_	+	+	—	—
*P*_*lag*1*,lag*2_	76, 1	51, 16	10, 1	7, 3
*H*_0*,*0_	+	+	—	—
*H*_*lag*1*,lag*2_	106, 23	38, 1	15, 2	4, 1
*W*_0*,*0_	+	—	—	—
*W*_*lag*1*,lag*2_	—	119, 86	—	17, 12
AIC	8710.764	7866.722	1083.408	980.019
*r*_*S*_ - training	0.817	0.885	0.899	0.912
*r*_*S*_ - test	0.870	0.876	0.891	0.899
RMSE - training	2.302	1.892	1.693	1.259
RMSE - test	2.475	2.229	1.960	1.752

Included parameters of the *CCM* and *OPT* model indicated by”+” or the used lag (not included quantities indicated by”—”), as well as their AIC, correlation coefficient *r*_*S*_ and root mean square error *RMSE*.

To test whether those regression models can be used to predict *Cx. pipiens/restuans* population dynamics we calculated Spearman’s rank correlation coefficient *r*_*S*_ (as the data were non-Gaussian distributed) and the root mean square error *RMSE* between the model predictions and the observations. All statistical analyses were conducted with the freely available statistical computing environment R [28]. The package genalg [29] was used to conduct the genetic algorithm.

## Results

### Cross-correlation maps

The *Cx. pipiens/restuans* capture rates were positively correlated with daytime length, temperature and precipitation (Figure 3). Humidity and wind speed were negatively correlated with mosquito abundance. The highest correlation was found for daytime length averaged from the fifth and fourth week prior the capture event with *r*_*S*_ (5*,* 4) = 0*.*898 (daily *r*_*S*_ (39*,* 28) = 0*.*873). Mosquito abundances were highly correlated with the mean temperature of the 2 weeks prior a capture event resulting in *r*_*S*_ (2*,* 1) = 0*.*870. The highest daily correlation was estimated for the average temperature calculated from the last 18 days prior the capture day with *r*_*S*_ (18*,* 1) = 0*.*853. Of the tested environmental quantities precipitation showed the weakest association with mosquito abundance. The highest correlation for this quantity was found for precipitation averaged over the 10 weeks prior to capture with *r*_*S*_ (10*,* 1) = 0*.*487 (daily data: *r*_*S*_ (76*,* 1) = 0*.*474). Interestingly, although precipitation was positively correlated with mosquito capture rates, humidity showed to be negatively correlated with mosquito capture rates. The highest effect was found for the humidity averaged over the time period of 15 to 2 weeks prior capture resulting in *r*_*S*_ (15*,* 2) = *−*0*.*561 (daily data: *r*_*S*_ (106*,* 23) = *−*0*.*562). A maximum negative correlation of *r*_*S*_ (3*,* 1) = *−*0*.*621 (daily *r*_*S*_ (23*,* 1) = *−*0*.*647) was found for wind speed averaged over 3 weeks (23 days) prior to capture.

### Regression analysis

**Figure 4:**
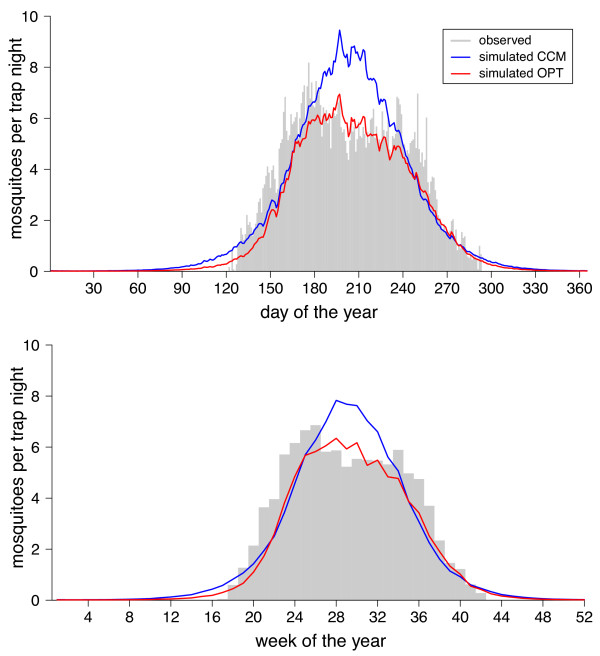
**Average annual *Cx. pipiens/restuans *abundance.** Average seasonal cycle of observed (bars) versus predicted (lines) daily and mean weekly mosquitoes trapped in Cook County.

**Figure 5:**
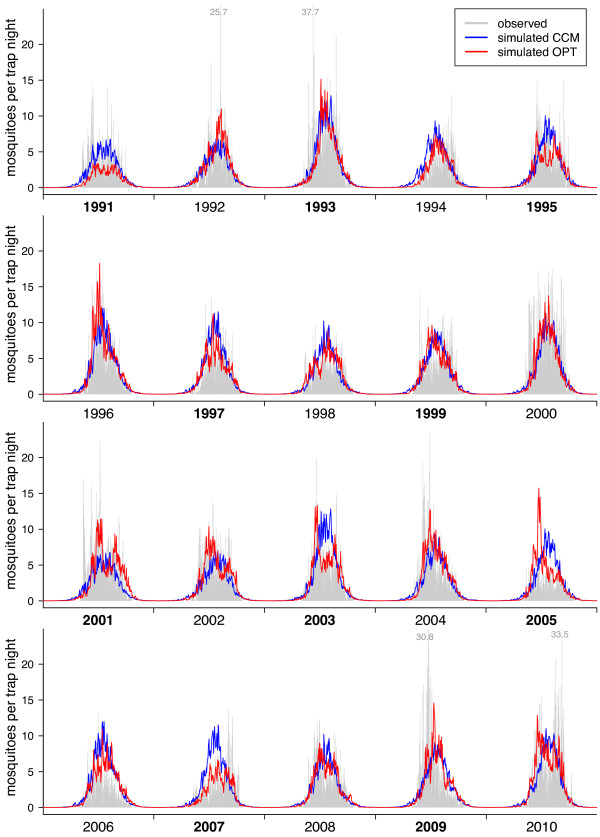
**Daily dynamics of the *Cx. pipiens/restuans *abundance.** Daily number of *Cx. pipiens/restuans* females per trap night. Gray bars represent daily capture rates, lines the predictions from the models. Years of the test data are marked with bold characters.

**Figure 6:**
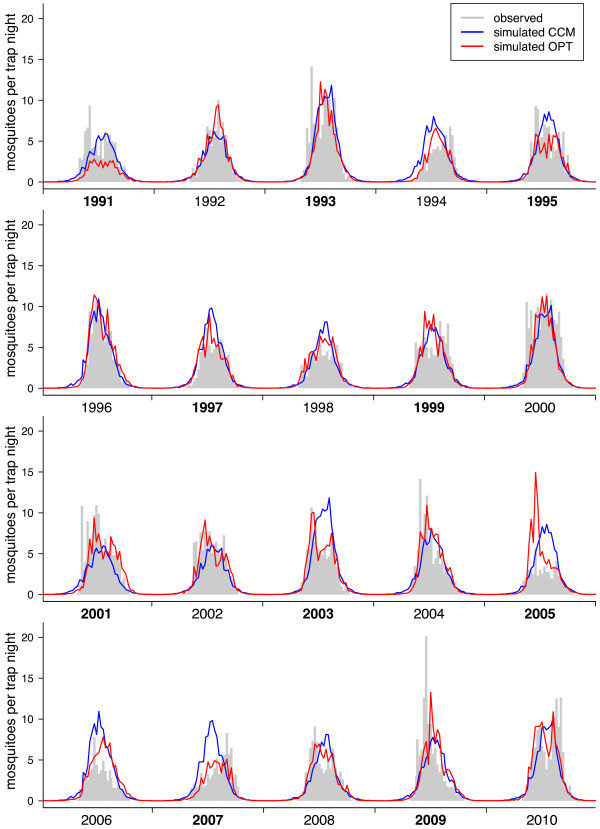
**Weekly dynamics of the Cx. pipiens/restuans abundance.** Weekly means of *Cx. pipiens/restuans* females per trap night. Gray bars represent mean weekly capture rates, lines the predictions from the models. Years of the test data are marked with bold characters.

The *CCM* model calculated for the daily data set included all possible environmental quantities, with the exception of lagged wind speed (Table 1). The *CCM* model for the weekly data was a much more reduced model, containing only the temperature during the capture week and the lagged quantities for daytime length, temperature, precipitation and humidity. To test whether the information gained from the CCMs could be used to predict the average seasonal cycle of female *Cx. pipiens/restuans*, an average mosquito year for both weekly and daily data, respectively, was compiled. The model predictions reproduced the average seasonal cycle of the mosquito abundances fairly accurately (Figure 4). The correlation of the observed mean seasonal cycle with the *CCM* model was *r*_*S*_ = 0*.*899 for the daily and *r*_*S*_ = 0*.*917 for the weekly data set. Similar results were obtained for the entire 20 years time series (Figures 5 and 6).

The *OPT* model obtained by the genetic algorithm included similar environmental quantities (Table 1).

Temperature and precipitation time lags were within the frame obtained by the CCMs, but span a shorter time period. The number of captured mosquitoes increased with temperature at the capture day, but there was also a negative effect with temperature shortly before the capture (Table 2). Thus, a higher temperature at the time of capture compared to the temperature at the lagged time interval results in higher capture rates than the other way around. Daytime length at the time of the capture and 13 weeks prior to the capture event were positively associated with the *Cx. pipiens/restuns* abundance, causing a shift in the seasonal cycle between the daytime length and the mosquito abundance of about 4 weeks. The lag for wind speed included in the *OPT* model was from rather far back in time. It comprises a time frame of about 3 to 4 months before the capture event. The time span in which humidity influenced the capture rates was shifted closer to the capture event and also enfolds the much narrower time span of approximately the month preceding the capture. Using the *OPT* model estimates obtained by fitting the model from to the training data set (Table 2), the mean seasonal cycle of the mosquito abundance as well as the mosquito dynamics of the last 20 years were predicted. The correlations of the observed versus predicted mean seasonal abundance resulted in *r*_*S*_ = 0*.*906 for the daily and *r*_*S*_ = 0*.*922 for the weekly data set (Figure 4). They were also well adapted to predict the course of the mosquito population over the last 20 years (Figures 5 and 6).

**Table 2:** Model summary

	***β***	**SE**	**z value**	**p**
**Daily data**				
**Intercept**	−**22.3142**	**0.5200**	−**42.9118**	**< 0.0001**
*D*_0*,*0_	1.0901	0.0281	38.8183	*<* 0.0001
*D*_85*,*85_	0.6453	0.0216	29.8125	*<* 0.0001
*T*_0*,*0_	0.0441	0.0037	11.8177	*<* 0.0001
*T*_12*,*1_	−0.0751	0.0065	−11.5170	*<* 0.0001
*P*_0*,*0_	0.0044	0.0013	3.4509	0.0006
*P*_51*,*16_	0.0990	0.0081	12.2828	*<* 0.0001
*H*_0*,*0_	−0.0131	0.0013	−10.2594	*<* 0.0001
*H*_38*,*1_	0.0453	0.0027	17.0574	*<* 0.0001
*W*_119*,*86_	−0.5238	0.0325	−16.1039	*<* 0.0001
**Weekly data**				
Intercept	−22.4553	1.4451	−15.5389	*<* 0.0001
*D*_0*,*0_	1.1318	0.0820	13.8066	*<* 0.0001
*D*_13*,*13_	0.6034	0.0551	10.9537	*<* 0.0001
*T*_0*,*0_	0.0386	0.0150	2.5816	0.0098
*T*_1*,*1_	−0.0612	0.0148	−4.1208	*<* 0.0001
*P*_7*,*3_	0.1036	0.0208	4.9686	*<* 0.0001
*H*_4*,*1_	0.0354	0.0061	5.7550	*<* 0.0001
*W*_17*,*12_	−0.5573	0.0940	−5.9315	*<* 0.0001

Summary of the regression model after the parameter optimization of the lags (*OPT*) from the daily and weekly data (trainings data set). For each quantity the regression coefficient *β* and the standard error of the regression coefficient SE, the test statistic (z value) and the p-value is given. The used environmental quantities were D - daytime length, T - temperature, P - precipitation, H - relative humidity, and W - wind speed. The subscript 0,0 represents the environmental quantity at the time of capture. Two subscripts indicate the beginning and the end of the lagged time period.

Both, the *CCM* and the *OPT* model, were able to predict the beginning and the end of the seasonal cycle as well as the inter-annual differences in the amplitude correctly. However, the models produced by the genetic algorithm are better adapted to predict mosquito abundances during mid-summer as they could reproduce the bimodal seasonal peak abundance of some of the years. The advantage of using the optimized time lags obtained by the genetic algorithm compared to implementing the results from the CCMs directly into a regression model is shown by the higher correlation coefficient and lower *RMSE* (both for the training as well as the test data sets) and by a significantly improved AIC (Table 1).

## Discussion

Temperature dependent life expectancy and development rates make it difficult to distinctly assign the time lags influencing mosquito capture rates to the different developmental stages. Under summer field conditions adult *Culex spp.* have a mean lifespan of about 1 week [30,31]. Weather conditions within this period therefore have their main impact on adult mosquitoes. The duration of the aquatic stages also varies with temperature, and weather conditions from about 8 to 20 days prior to capture presumably affect the aquatic stage [7]; weather conditions from about 20 to 28 days affect the adult stage of the parent generation. Previous studies with CCMs considered a time period of about 4 weeks, which approximately represents the lifetime of a mosquito, inclusive all aquatic stages [5,18,19]. The results of this study however show that environmental factors have to be considered further back in time. As this exceeds the mean mosquito lifespan, it is likely that weather conditions occurring far back in time do not affect the current population directly, but affect previous generations. Due to exponential growth rates even small effects of weather conditions on a mosquito population could therefore result in vast effects in future generations.

Daytime length may be the most important factor generating the seasonal pattern of mosquito abundance as it regulates - together with temperature - the incidence of diapause. The conditions occurring during pupal development have been shown to determine whether the adult female undergoes diapause [11,32]. The results of the regression models revealed that maximum mosquito abundance was reached 4 weeks after the longest daytime length. This is also reflected by the results of the CCMs. The shift of 4 weeks between the photoperiod and the mosquito abundance peak may indicate that effects on the pupal stage of the parent generation may be more influential than effects on the current generation.

The time with the strongest effect of ambient temperature was at the time of the capture and the time shortly before this event, indicating that mostly adult mosquitoes were affected. Mosquito capture rates increased with increasing temperature, as already shown in previous studies [5,6]. Furthermore, it has been demonstrated that considerable changes in the temperature at the capture event compared to the previous days affect the number of captured mosquitoes. Chuang et al. [13] found a similar temperature effect for *Cx. tarsalis* and *Ae. vexans* as the authors described a positive influence of temperature at the week of the capture and a lesser negative effect of temperature with a 2 week lag. Thus it seems that female *Cx. pipiens/restuans* are strongly influenced by temperature changes. This might indicate that they delay flight activities (e.g. host searching) until more favorable temperature conditions occur and considerably decrease their activities at a sudden temperature drops. Sudden temperature changes could not only affect their activity, but could also possibly influence their survival rates.

Capture rates were influenced by rainfall accumulated over long time periods exceeding the typical mosquito life span. This indicates that the amount of precipitation during the previous generation had a stronger effect on the capture rates than the rain falling during the lifespan of the captured mosquitoes. As many of the potential breeding sites, such as shallow temporal ponds, only exist after a certain amount of rainfall, the increased number of breeding sites after rainfall had a positive effect on the number of captured mosquitoes weeks later. Those pools need to be sustained by rainfall for several weeks to ensure the survival of the aquatic stages. At our study site, the suburban area of Chicago, catch basins represent an important breeding site for mosquitoes [14,33]. In temporary as well as in stagnant water bodies rainfall increases the water volume. This causes a decreasing larval density, which results in increased development rates and decreased mortality rates [34,35]. Previous studies have shown that high amounts of rainfall decrease the number of mosquito larvae in catch basins dramatically [14,36]. Interestingly, this negative effect of precipitation on the larvae was not visible in our study where the effects of previous rainfall on the number of adult mosquitoes was investigated. It is possible that this negative effect on larvae, which is noticeable for about 4 days [36], was overlain by the subsequent longer lasting positive effects mentioned above.

In contradiction to the results from the CCMs, a high relative humidity in the month prior the capture event had a positive effect on mosquito capture rates. This shows that interrelations between environmental quantities may shroud the effect of one quantity on the mosquito capture rates when it is considered without others (as it was done in the CCMs). The regression analysis on the contrary allows to control for the effect of the other quantities included in the model. This positive effect of relative humidity was also found for several Culicidae species, showing that relative humidity influences mosquito activity patterns and the dynamics of oviposition [15,37].

Experiments by Hoffmann [38] indicate that a high wind speed does not reduce flight activity in mosquitoes, but rather impairs the mosquito orientation by deluding attracting stimuli like CO_2_ and thus reducing capture rates. The results of this study indicate that the effects of wind on the parent generation may have a more important effect on the capture rates than on the current generation. The negative effect of elevated wind speed in the several weeks prior to the capture event may be caused by a lower chance for a blood meal during this time period.

Mosquito larvae control strategies conducted by the mosquito control agency in the study area were not accounted for in this study. Those strategies have been changed over the course of 20 years in the methods used as well as in efficiency. Aberrations of our model results from the observation data could thus be caused by changes in the used mosquito larvae control strategies. For further applications of the presented models one has to keep in mind that they represent a “managed” *Cx. pipiens/restuans* population. This is on the one hand a benefit, as in many inhabited areas (in the U.S.A.) there is some kind of mosquito control, especially in endemic areas of mosquito borne diseases like WNV. On the other hand, one has to be careful when adopting this model for other regions.

Cross-correlation maps have been proven to be useful tools in investigating time lagged associations between vector abundance and environmental factors [5,18-20]. However, as pointed out by Cohnstaedt [39], there are two major disadvantages of CCMs. First, they do not consider that fact that adults at one time period are a function of the number of adults from the previous generation. And second, that lagged weather variables by a fixed time period ignores the temperature dependence of developmental rates. The first argument concurs with the results of this study. By extending in this study the maximum time lag we were able to reveal that environmental effects on previous generations are likely more important factors describing the number of captured mosquitoes than the effect of those variables on the current generation. With our analyses we are not able to counter the second argument, we can just be careful when interpreting the results found regarding their association to different developmental stages.

## Conclusions

The final question is, whether the information gained from CCMs could be used to predict *Cx. pipiens/restuans* population dynamics. The applicability of the models for daily and weekly predictions depends of course on ones expectations. On both time scales the models resulted in a good predictability of the seasonal cycle. Interannual differences in the mosquito abundance could in large part be reproduced. Especially the optimized model for the weekly data allowed to predict the mosquito abundances to a high degree, and could be of practical use, e.g. planning of mosquito control strategies and simulation of mosquito borne diseases.

## Competing interests

The authors declare that they have no competing interests

## Author’s contributions

The project was designed by FR and KL, the data analyzed by KL and KB, and the paper written by KL and FR. All authors read and approved the final version of the manuscript.

## References

[B1] Farajollahi A Fonseca DM Kramer LD Marm Kilpatrick A “Bird biting” mosquitoes and human disease: a review of the role of Culex pipiens complex mosquitoes in epidemiology Infect Genet Evol 2011 11 7 1577 1585 21875691 10.1016/j.meegid.2011.08.013 PMC3190018

[B2] Harrington LC Poulson RL Considerations for accurate identification of adult Culex restuans (Diptera: Culicidae) in field studies J Med Entomol 2008 45 1 8 18283935 10.1603/0022-2585(2008)45[1:cfaioa]2.0.co;2

[B3] Calisher CH Medically important arboviruses of the United States and Canada Clin Microbiol Rev 1994 7 89 116 8118792 10.1128/cmr.7.1.89 PMC358307

[B4] Kramer L Ebel G Dynamics of Flavivirus infection in mosquitoes Adv Virus Res 2003 60 187 232 14689695 10.1016/s0065-3527(03)60006-0

[B5] Chuang TW Ionides EL Knepper RG Stanuszek WW Walker ED Wilson ML Cross-correlation map analyses show weather variation influences on mosquito abundance patterns in Saginaw County, Michigan, 1989–2005 J Med Entomol 2012 49 4 851 858 22897045 10.1603/me11150

[B6] Wang J Ogden NH Zhu H The impact of weather conditions on Culex pipiens and Culex restuans (Diptera: Culicidae) abundance: a case study in peel region J Med Entomol 2011 48 2 468 475 21485391 10.1603/me10117

[B7] Madder DJ Surgeoner GA Helson BV Number of generations, egg production, and developmental time of Culex pipiens and Culex restauns (Diptera: Culicidae) in southern Ontario J Med Entomol 1983 20 3 275 287 6876091 10.1093/jmedent/20.3.275

[B8] Reisen WK Effect of temperature on Culex tarsalis (Diptera: Culicidae) from the Coachella and San Joaquin valleys of California J Med Entomol 1995 32 5 636 645 7473618 10.1093/jmedent/32.5.636

[B9] Rueda LM Patel KJ Axtell RC Stinner RE Temperature-dependent development and survival rates of Culex quinquefasciatus and Aedes aegypti (Diptera: Culicidae) J Med Entomol 1990 27 5 892 898 2231624 10.1093/jmedent/27.5.892

[B10] Vinogradova EB Culex pipiens pipiens mosquitos: taxonomy, distribution, ecology, physiology, genetics, applied importance and control 2000 Sofia: Pensoft Publishers

[B11] Spielman A Wong J Environmental control of ovarian diapause in Culex pipiens Ann Entomol Soc Am 1973 66 4 905 907

[B12] Wilton DP Smith GC Ovarian diapause in three geographic strains of Culex pipiens (Diptera: Culicidae) J Med Entomol 1985 22 5 524 528 4045946 10.1093/jmedent/22.5.524

[B13] Chuang TW Hildreth MB Vanroekel DL Wimberly MC Weather and land cover influences on mosquito populations in Sioux Falls, South Dakota J Med Entomol 2011 48 3 669 679 21661329 10.1603/me10246 PMC3117223

[B14] Geery P Holub R Seasonal abundance and control of Culex spp. in catch basins in Illinois J Am Mosq Control Assoc 1989 5 4 537 540 2614404

[B15] Chaves LF Kitron UD Weather variability impacts on oviposition dynamics of the southern house mosquito at intermediate time scales Bull Entomol Res 2011 101 6 633 641 21208506 10.1017/S0007485310000519

[B16] Strickman D Rate of oviposition by Culex quinquefasciatus in San Antonio, Texas, during three years J Am Mosq Control Assoc 1988 4 339 344 3199124

[B17] Buckner EA Blackmore MS Golladay SW Covich AP Weather and landscape factors associated with adult mosquito abundance in southwestern Georgia, U.S.A J Vector Ecol 2011 36 2 269 278 22129398 10.1111/j.1948-7134.2011.00167.x

[B18] Curriero FC Shone SM Glass GE Cross correlation maps: a tool for visualizing and modeling time lagged associations Vector Borne Zoonotic Dis 2005 5 3 267 275 16187896 10.1089/vbz.2005.5.267

[B19] Shone SM Curriero FC Lesser CR Glass GE Characterizing population dynamics of Aedes sollicitans (Diptera: Culicidae) using meteorological data J Med Entomol 2006 43 2 393 402 16619626 10.1603/0022-2585(2006)043[0393:cpdoas]2.0.co;2

[B20] Walsh AS Glass GE Lesser C Curriero FC Predicting seasonal abundance of mosquitoes based on off-season meteorological conditions Environ Ecol Stat 2008 15 3 279 291

[B21] Brugger K Rubel F Bluetongue disease risk assessment based on observed and projected Culicoides obsoletus spp. vector densities PLoS One 2013 8 4 e60330 23560090 10.1371/journal.pone.0060330 PMC3613389

[B22] Homer C Huang C Yang L Wylie B Coan M Development of a 2001 national land-cover database for the United states Photogramm Eng Rem S 2004 70 7 829 840

[B23] NNCD climate data online http://www7.ncdc.noaa.gov/CDO/cdoselect.cmd

[B24] Forsythe WC Rykiel EJ Stahl RS Wu H Schoolfield RM A model comparison for daylength as a function of latitude and day of year Ecol Model 1995 80 87 95

[B25] Kottek M Grieser J Beck C Rudolf B Rubel F World map of the Köppen-Geiger climate classification updated Meteorol Z 2006 15 3 259 263

[B26] Akaike H A new look at the statistical model identification IEEE T Automat Contr 1974 19 6 716 723

[B27] Hamblin S On the practical usage of genetic algorithms in ecology and evolution Methods Ecol Evol 2013 4 2 184 194

[B28] R Development Core Team: R A language and environment for statistical computing, version 2.15.2 2012 Vienna, Austria: R Foundation for Statistical Computing

[B29] Willighagen E Genalg: R based genetic algorithm, version 0.1.1 2005 http://cran.r-project.org/web/packages/genalg/index.html

[B30] Elizondo-Quiroga A Flores-Suarez A Elizondo-Quiroga D Ponce-Garcia G Blitvich BJ Contreras-Cordero JF Gonzalez-Rojas JI Mercado-Hernandez R Beaty BJ Fernandez-Salas I Gonotrophic cycle and survivorship of Culex quinquefasciatus (Diptera: Culicidae) using sticky ovitraps in monterrey, northeastern Mexico J Am Mosq Control Assoc 2006 22 10 14 16646315 10.2987/8756-971X(2006)22[10:GCASOC]2.0.CO;2

[B31] Reisen WK Milby MM Presser SB Hardy JL Ecology of mosquitoes and St. Louis encephalitis virus in the Los Angeles Basin of California, 1987–1990 J Med Entomol 1992 29 4 582 598 1495066 10.1093/jmedent/29.4.582

[B32] Eldridge BF The effect of temperature and photoperiod on blood-feeding and ovarian development in mosquitoes of the Culex pipiens complex AmJTrop Med Hyg 1968 17 133 140 10.4269/ajtmh.1968.17.133 5688903

[B33] Crans W A classification system for mosquito life cycles: life cycle types for mosquitoes of the northeastern United States J Vector Ecol 2004 29 1 10 15266736

[B34] Buth JL Brust RA Ellis RA Development time, oviposition activity and onset of diapause in Culex tarsalis, Culex restuans and Culiseta inornata in southern Manitoba J Am Mosq Control Assoc 1990 6 55 63 1969927

[B35] Olejnicek J Gelbic I Differences in response to temperature and density between two strains of the mosquito, Culex pipiens molestus Forskal J Vector Ecol 2000 25 2 136 145 11217212

[B36] Gardner AM Hamer GL Hines AM Newman CM Walker ED Ruiz MO Weather variability affects abundance of larval culex (Diptera: Culicidae) in storm water catch basins in suburban Chicago J Med Entomol 2012 49 2 270 276 22493843 10.1603/me11073 PMC4053168

[B37] Dow RP Gerrish GM Day-to-day change in relative humidity and the activity of Culex nigripalpus (Diptera: Culicidae) Ann Entomol Soc Am 1970 63 4 995 999 5449406 10.1093/aesa/63.4.995

[B38] Hoffmann EJ Miller JR Reassessment of the role and utility of wind in suppression of mosquito (Diptera: Culicidae) host finding: stimulus dilution supported over flight limitation J Med Entomol 2003 40 5 607 614 14596273 10.1603/0022-2585-40.5.607

[B39] Cohnstaedt LW Rochon K Duehl AJ Anderson JF Barrera R Su NY Gerry AC Obenauer PJ Campbell JF Lysyk TJ Allan SA Arthropod surveillance programs: basic components, strategies and analysis Ann Entomol Soc Am 2012 105 2 135 149 10.1603/AN11127 PMC4630213 26543242

